# gDNA extraction yield and methylation status of blood samples are affected by long-term storage conditions

**DOI:** 10.1371/journal.pone.0192414

**Published:** 2018-02-07

**Authors:** Charlotte Schröder, Werner Steimer

**Affiliations:** Institut für Klinische Chemie und Pathobiochemie, Klinikum rechts der Isar, Technische Universität München, München, Germany; Centre de Recherche en Cancerologie de Lyon, FRANCE

## Abstract

Epigenetics is believed to provide great chances for a better understanding of the development and treatment of many diseases where the analysis of genomic DNA has so far failed to provide conclusive answers. Methylcytosine is a frequently used quantitative marker of epigenetic studies. Since immediate analysis of sampled material is in most cases not possible, storage time and conditions are critical aspects regarding the quality of genomic DNA and reliability of analysis. Blood is frequently used for such analyses. We, therefore, collected blood samples of ten volunteers and stored them under various conditions for ten months: -70°C, -20°C, 2–8°C and room temperature. An additional aliquot was frozen at -70°C and thawed once a week at room temperature. We then compared the DNA extraction yields and methylation status in relation to storage time and conditions. We found significantly lower DNA extraction yields (up to -97.45%; p ≤ 0.001) as well as significantly higher methylation levels after ten months of storage (up to +42.0%; p ≤ 0.001). These results suggest that storage time has an important influence on DNA analyses of blood samples for all storage conditions. This might be due to differences in stability of methylated and non-methylated DNA. Our study indicates that storage conditions and time may be a critical factor for epigenetic methylation studies and require rigorous validation. For reliable analyses we, therefore, recommend to perform epigenetic analysis directly after sample collection.

## Introduction

Epigenetic studies have become widespread and are continually gaining in importance. Researchers believe that epigenetics will in future provide the new insights into the development and the treatment of diseases which genetics was not able to explain so far [1, 2]. Nowadays, methylcytosine is the key molecule in epigenetics and serves as a quantitative marker which changes throughout lifetime [3]. Consequently, stable sample material is essential for the reliability of analytical results of epigenetic studies. The most frequently used sample material is human whole blood, from which genomic DNA (gDNA) is extracted. However, immediate gDNA extraction and analysis is often not viable. This may be due to economic reasons, e.g. if several samples are collected for analysis over a longer period of time. Other reasons may be time or logistic constraints. Thus, blood samples are often stored for up to several years. This is mostly done in deep freeze at -80°C [4]. However, this is not always possible, for instance if the sample material has to be transported over long distances. It is, therefore, essential to determine the impact of storage conditions on gDNA methylation in order to ensure the reliability of epigenetic studies.

Possible degradation of gDNA as a result of the storage of the sample material might greatly interfere with the results of molecular biology testing. The determination of methylcytosine as a quantitative marker may be particularly susceptible to this. Several studies report poorer gDNA yields during extraction after prolonged storage time of blood samples [5–7]. However, it is unclear whether this is also associated with poorer DNA quality. Studies suggest that gDNA can still be used for numerous molecular biology studies and provides reliable test results [6, 8]. In contrast, other studies have observed a change in gDNA quality after as little as a few days of storage [9, 10]. To date, not much is known regarding the storage stability of methylcytosine. While it is believed that gDNA stored at -80°C can be used for molecular biology experiments for many years, so far only one study has investigated the changes in methylation levels of gDNA extracted from blood samples after storage for up to one year [11]. However, this study investigated overall methylation levels of whole gene sequences by quantitive PCR.

The aim of this study was, therefore, to determine the influence of different storage conditions and periods on gDNA methylation of single CpG sites with the goal of ensuring the reliability and comparability of epigenetic studies. Therefore, we used next generation sequencing which shows greater precision than quantitive PCR [12, 13].

## Methods

This preanalytic validation was part of a larger study assessing pharmacogenetics in psychiatric patients, which has been approved by the local ethics committee at the “Klinikum rechts der Isar der Technischen Universität München” and is in accordance with the current revision of the Helsinki Declaration. Patients were informed of the aims of the study and gave written informed consent which could be withdrawn at any time. For the study mentioned above EDTA blood was collected and stored for diverse periods of time. In order to analyze methylation levels accurately, we, therefore, investigated in this study if methylation levels of these samples remain stable and are comparable despite varying long term storage conditions. We stored EDTA blood samples from ten volunteers for a total of ten months under various conditions. The blood was taken at the Institute of Clinical Chemistry and Pathobiochemistry at the Klinikum rechts der Isar, Munich, Germany on December 14–16^th^ 2015. One aliquot was extracted and sequenced immediately after blood collection (start time T_0_). Additional aliquots were stored at -70°C, -20°C, at 2–8°C and at room temperature (20–25°C). Another aliquot was frozen at -70°C and thawed once a week at room temperature. Analyses were carried out after one (T_1_), three (T_3_) and ten months (T_10_) of storage by sequencing an amplicon of 208 base pairs (bp) in the intron 1 of the HIF3A gene comprising a total of ten CpG sites. We focused on this amplicon and investigated the stability of the methylation pattern in this gene segment, due to our interest in this region in the context of the study mentioned above.

### gDNA extraction and extraction yields

First, gDNA was extracted from 300 μl of whole EDTA blood using the Wizard Genomic DNA Purification Kit (Promega, Madison/USA). If no white cell pellet was visible following cell lysis and subsequent centrifugation, this step was repeated. The gDNA content was determined by spectrofluorometry using Qubit (Thermo Fisher Scientific, Waltham/USA).

### gDNA Methylation

Genomic DNA methylation was measured by means of bisulfite conversion using the EZ Methylation Lightning Kit (Zymo Research, Irvine/USA) and subsequent pyrosequencing on the GS Junior (Roche, Basel/Switzerland) according to the manufacturer’s instructions. For library preparation, we used the forward primer GTTTTGGGTTTAATAAGGAATTTTATTT and the reverse primer RATACAACCAAAACCCRAATAC (R = A/G). We attached the manufacturer’s GS Junior recognition sequences and multiplex identifiers (MIDs) to these sequence specific primers in order to identify the individual samples. For analysis, we used the Amplicon Variant Analysis (AVA) software 3.0 (Roche). Due to the manufacturer’s advice, the short quality filter setting was increased from the default 4 allowed failures per 320 bp to 18 allowed failures per 168 bp. All samples were sequenced at least twice. An overview of the used primers and MIDs, as well as the pipetting scheme and PCR program for the library preparation, is given in S1 Supporting Information.

To validate the method, gDNA standards with known methylation status were sequenced (Human WGA Methylated & Non-methylated DNA Set [Zymo Research]). Methylated and non-methylated gDNA were mixed to provide samples with the following methylation levels: 0, 25, 50, 75 and 100%. The results of the standard measurements are available in S1 Supporting Information.

### Statistics

Statistical analyses were performed via SPSS 21 (IBM, Armonk/USA). Normal distribution was estimated according to Kolmogorov-Smirnov, Shapiro-Wilk test and boxplot. We used the paired t-test to test for diversity of samples. A p-value of p ≤ 0.05 was considered to be significant. To test for agreement of samples we created Bland-Altman plots. Graphics were generated with SPSS 21 or MS EXCEL 2010 (Microsoft Corporation, Seattle/USA).

## Results

### Extraction yields

**Fig 1:**
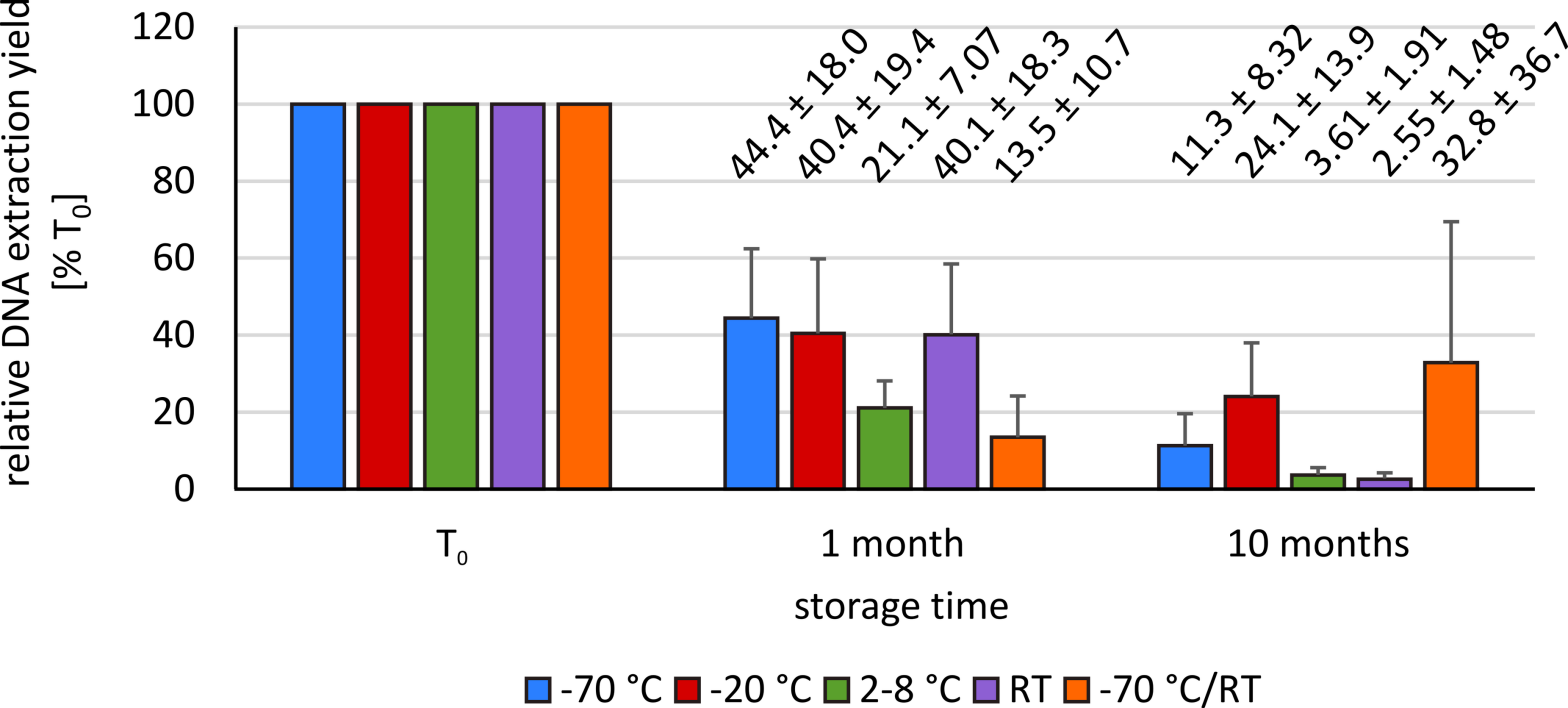
Mean relative gDNA extraction yields and standard deviations (SD) according to different storage conditions and storage times. The relative values were calculated with regard to the gDNA extraction yield at T_0_ directly after sample collection: The gDNA yields for all storage conditions were decreasing. After ten month of storage the yields of gDNA for all storage conditions were significantly less compared to time T_0_ (p ≤ 0.001).

For all storage conditions, the yield of gDNA after the extraction decreased in line with storage time (Fig 1). After ten months of storage, it was difficult to extract sufficient gDNA for bisulfite conversion and sequencing. Therefore, the cell lysis step of the gDNA extraction protocol had to be repeated in order to obtain an adequate amount of material. However, extraction yields for all storage conditions after ten months were significantly lower compared to gDNA yield obtained directly after blood collection (mean gDNA extraction yield [T_10_]: 2.55%–32.8%; p < 0.001). Only the frozen samples which were thawed once per week (-70°C/RT) showed higher extraction yields after ten months (13.5% [T_1_] vs. 32.8% [T_10_]). However, standard deviations (SD) of measurements based on these samples were high (mean SD_-70°C/RT_ = ± 36.7). Differences in extraction yield for samples stored at -70°C, -20°C and samples which were thawed once per week on one hand and for samples stored at 2–8°C and 20–25°C on the other hand were statistically significant (0.002 < p < 0.038). The T-test did not show any significant differences for samples stored at -70°C, -20°C and samples which were thawed once per week as well as for samples stored at 2–8°C and 20–25°C (0.106 < p < 0.556; p = 0.360).

### gDNA methylation

**Fig 2:**
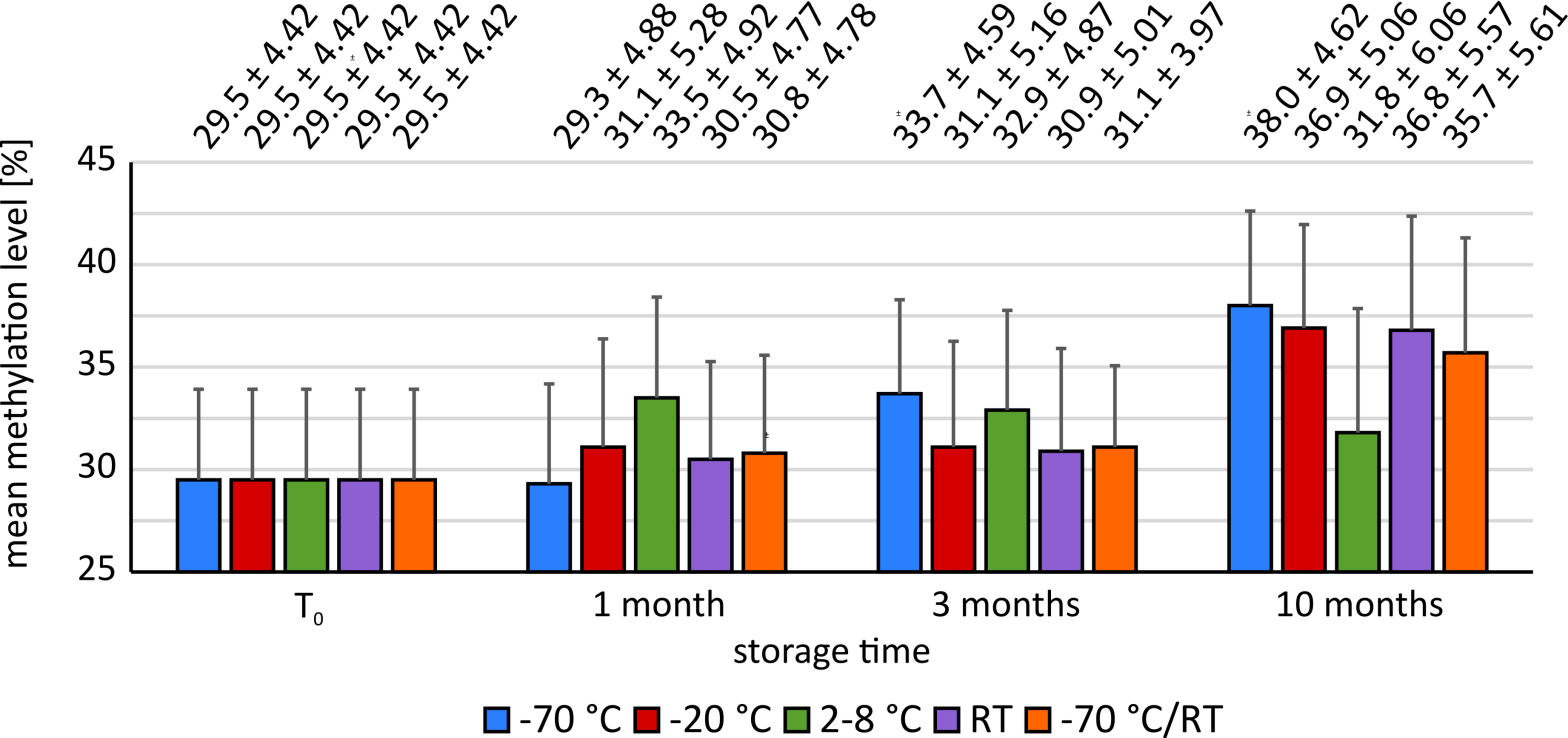
Mean methylation levels ± SD for the sum of all CpG sites at the start time T_0_ and after storage for one, three and ten months. Methylation status for all storage conditions are significantly increasing with storage time. Only blood samples stored at -70°C for one month show in mean lower methylation levels compared to T_0_, however, this correlation was not significant.

Methylation was determined directly after blood collection (T_0_) and after one month (T_1_), three months (T_3_) and ten months (T_10_) of storage. An overview of the mean methylation levels and absolute changes of methylation compared to T_0_ is shown in Table 1 and Fig 2. The values represent mean values of all ten measured CpG sites. In most cases, T-test showed significant increases in methylation levels after one, three and ten months compared to methylation analysis directly after blood collection. The highest values were reached after ten months of storage, except for storage at 2–8°C where the maximum methylation level was observed after one month. The progress of methylation levels of the samples frozen at -20°C, stored at room temperature and of the samples thawed once a week was almost identical. Here, the methylation levels increased only slightly after one and three months, while a very significant increase was observed after ten months. In contrast, the samples frozen at -70°C showed a lower methylation level after one month. After three and ten months, methylation levels for these samples were the highest compared to the other storage conditions. However, methylation levels for these four storage conditions after ten months differ only within 2.3% with a standard deviation between 4.62 and 5.61. Thus, we considered the increase of these methylation levels as to be equal. For the samples stored in the refrigerator (2–8°C), the methylation levels showed a less pronounced but still significant increase compared to the other storage conditions. Here, the maximum was reached after one month, after which the methylation level decreased again. The mean values of all ten CpG sites were representative for each individual CpG site. In general, measured methylation levels increased with longer storage times. Especially after ten months, methylation was higher for all storage conditions. This effect reached significance in most cases (0.000 < p < 0.037) (for further details see S1 Supporting Information). We further tested for differences in increase in methylation between individual CpG sites. Increase in methylation was significantly different (p < 0.001). Comparison showed the tendency that CpG sites with low methylation levels (< 20%; CpG 1, 3, 4, 5, 7) gained more methylation relating to their basic levels (mean percentage methylation increase = 43%). CpG sites with high methylation levels (> 20%; CpG 2, 6, 8, 9, 10) showed less percentage increase of methylation (mean percentage methylation increase = 29%).

**Table 1:** Overview of the mean methylation levels and absolute changes (dif abs.) in methylation compared to T_0_ for the sum of all CpG sites after storage for one, three and ten months as well as p-values. Methylation levels for all storage conditions are significantly increasing with storage time. Only blood samples stored at -70°C for one month show in mean lower methylation levels compared to T_0_, however, this correlation was not significant.

storage conditions	T_0_	T_1_	dif abs. 1 M	p	T_3_	dif abs. 3 M	P	T_10_	dif abs. 10 M	p
**-70°C**	29.5	29.3	-0.179	0.524	33.7	+4.20	<0.001*	38.0	+8.52	<0.001*
**-20°C**	29.5	31.1	+1.61	<0.001*	31.1	+1.62	0.001*	36.9	+7.40	<0.001*
**RT**	29.5	30.5	+1.01	0.004*	30.9	+1.43	0.010*	36.8	+7.33	<0.001*
**2–8°C**	29.5	33.5	+4.03	<0.001*	32.9	+3.38	<0.001*	31.8	+2.33	0.001*
**-70°C/RT**	29.5	30.8	+1.32	<0.001*	31.1	+1.59	0.002*	35.7	+6.22	<0.001*

*p-values considered as significant (p ≤ 0.05)

**Fig 3:**
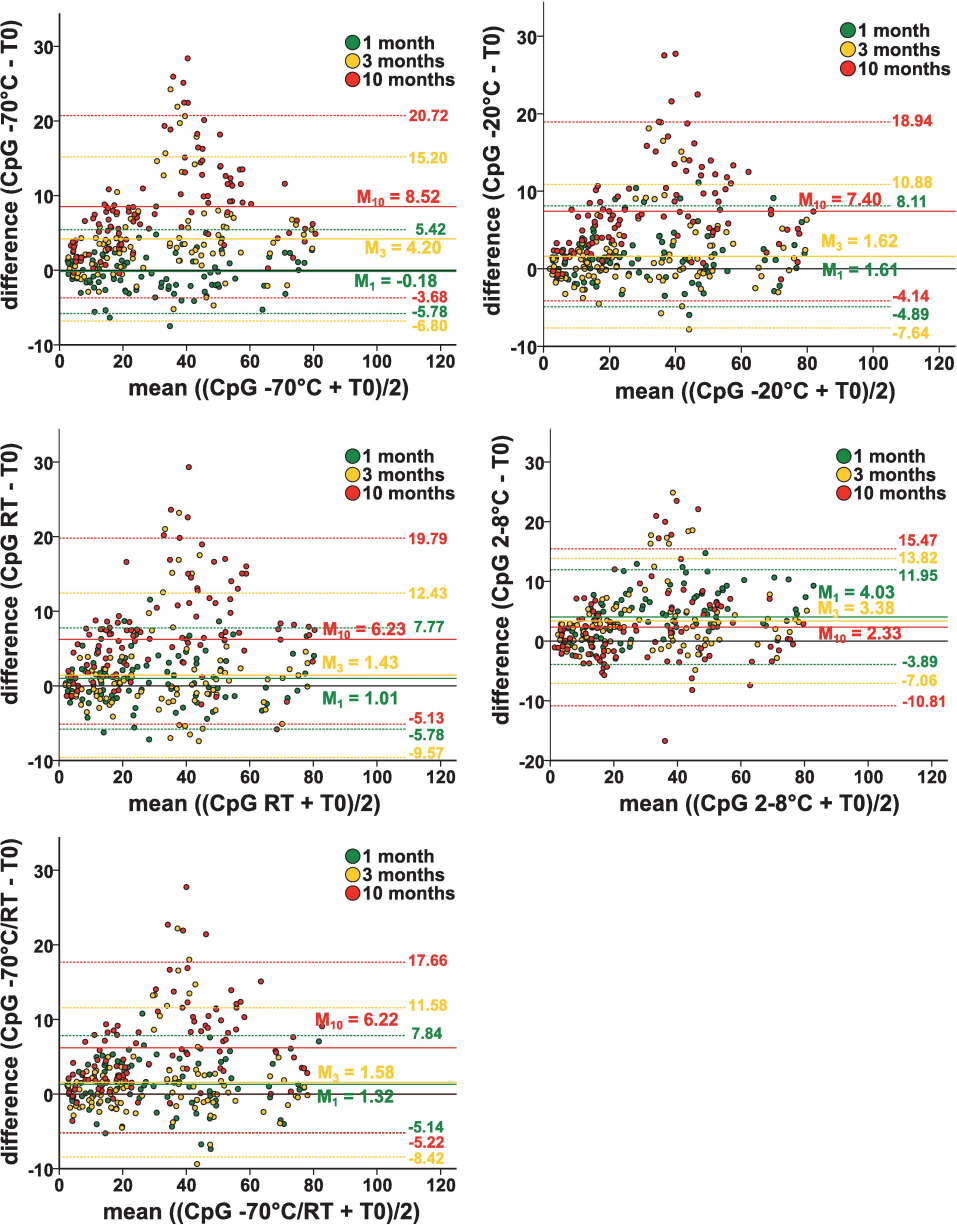
Bland-Altman plots of the measured methylation levels according to the different storage conditions. The differences between the measured values at the time T_1_ (green), T_3_ (yellow) and T_10_ (red) and the start time T_0_ and the corresponding mean are plotted. Further the mean differences (M; solid lines) and the 95% confidence intervals (M ± 2SD, doted lines) are shown. For all storage conditions there is a shift toward higher methylation levels after prolonged storage time.

Bland-Altman plots for the measured methylation values of the samples under different storage conditions differentiated by storage period are shown in Fig 3. This type of plot illustrates the variation for the differences of each single data pair and, therefore, allows to evaluate systematic bias and outliers [14, 15]. The differences between the measured values at time T_1_, T_3_ and T_10_ and the start time T_0_ are plotted against the corresponding mean values. Here, too, a shift occurs in all plots toward higher methylation levels after prolonged storage. This can also be seen for the samples stored at 2–8°C. While the mean value here does not indicate any change in methylation after ten months of storage, the observation of the individual values in the Bland-Altman plot also suggests a tendency towards higher methylation levels. To summarize, after one month of storage, the differences of the measured values T_1_-T_0_ still scatter uniformly around the mean value. After three months of storage, some outliers are emerging in the direction of higher methylation levels. After ten months, this effect is enhanced. In addition, for all storage conditions, an increase of the storage time goes in hand with an increase of the scattering of the measured value relative to the measurement at time T_0_ resulting in larger 95% confidence intervals.

## Discussion

### gDNA-yield

As part of this series of experiments, we were able to confirm previous studies with regard to poorer gDNA yields from EDTA blood after prolonged storage time [5–7]. Genomic DNA extraction yields continuously decreased over storage time. Generally the average yields after ten months for all storage conditions were less than one third compared to the blood samples analyzed at T_0_. For the samples thawed once per week particularly high variance in extraction yields was observed. When performing epigenetic studies, these results have to be taken into account. To overcome this issue, addition of preservative agents to the EDTA blood as recommended by Bulla et al. might be considered [11].

### gDNA methylation

However, it remains questionable whether the poorer gDNA yields are also associated with poorer gDNA quality. In several studies, decreased yields appeared to have no effect on gDNA quality and produced reliable results in a number of molecular biology tests [6, 8]. Likewise, Bulla et al. were unable to detect obvious degradation of gDNA by gel electrophoresis and long-range PCR [11]. By contrast, Malentacchi et al. observed changes in gDNA quality tested by the same methods only after a few days of storage of EDTA blood at 4°C [10]. Also Palmirotta et al. concluded from their investigations that storage has an impact on molecular biology test results and a stability study demonstrated progressive DNA degradation after repeated freeze and thaw cycles [9, 16]. We investigated if these influences of storage also have an impact on gDNA methylation.

Our results for the quantification of methylcytosine in intron 1 of the HIF3A gene suggest that storage of blood samples has a clear impact on the methylation level. A significant increase in methylation after ten months was measurable for all storage conditions. This increase was associated with lower gDNA yields in gDNA extraction. The Bland-Altman plots showed larger standard deviations and scattering of the measured differences for all samples with increasing storage time. Consequently, in the context of our study the quality of the gDNA appears to decrease over time. It, therefore, might be conceivable that methylated gDNA has a higher stability than non-methylated gDNA and thus might be less strongly degraded during storage and extraction. This hypothesis is supported by a study by Thalhammer et al., in which the authors examined the melting points and thus the stability of DNA double strands [17]. They found that methylated DNA had a higher melting point than non-methylated DNA and concluded that 5-methylcytosine stabilizes the DNA structure. However, this hypothesis disagrees with previous studies reporting methylated cytosine to be more susceptible to hydrolytic deamination and, therefore, to be less stable than non-methylated cytosine [18, 19]. A further explanation could be, that different proportions of cell types degrade at different rates in the blood. Therefore, there may be changes in the composition of different subtypes of leukocytes caused by storage. Since different cell types may have different methylation levels, the methylation values could also change due to altered cell ratios [20]. In this study, the concentration of cell types in the blood samples was not determined.

Bulla et al. also tested the methylation level of 22 selected genes from blood samples after storage for up to one year under various conditions [11]. In contrast to our results, they were unable to detect a significant change in gDNA methylation. Here, the absolute deviations were less than 1%. However, there were some methodological differences compared to our investigations. The authors did not consider single CpG sites but the methylation status of whole genes. Also, they determined methylation levels by methylation-sensitive and methylation-dependent enzymatic digestion and subsequent quantification by quantitive PCR. In several comparative studies, this method showed poorer precision and accuracy than pyrosequencing [12, 13]. Further studies in sperm and placenta tissue also did not show any change in methylation as a function of storage time. However, both studies only examined very short storage times of up to 72 and 24 hours, respectively [21, 22]. Further, other tissues, in particular sperm, are less prone to varying cell proportions over time than blood. This might be another explanation for these studies not to find any variance in methylation.

To summarize, this is, to our knowledge, the first study testing the stability of methylation patterns in stored EDTA blood samples by pyrosequencing. Our results indicate that gDNA degrades at all tested storage conditions leading to higher methylation levels with increasing storage time. This might be due to different stabilities of methylated and non-methylated gDNA or changes in the proportion of cell types. Fragmentation processes during storage as well as during the extraction process are possible reasons for lower gDNA yields during the extraction of gDNA from the blood samples.

One limitation of methylation analyses of whole blood is cell lysis during freezing which mixes up gDNA of different cell types. Previous studies have already identified differences in methylation due to the cell type [23, 24]. Therefore, methylation analyses of whole blood should count cell proportions and incorporate that as a confounding factor in their evaluation. In the context of methylation stability it would be interesting to analyze how isolated white blood cells or even extracted gDNA behave during long-term storage. Also, this would avoid interference due to different degradation rates of different cell types. Further experiments are needed to clarify this issue.

A difference in the stability of methylated and non-methylated gDNA in whole blood samples would represent a strong limitation for epigenetic analyses where a direct analysis of sample material is often not possible. Although in our study only ten individual CpG sites within one gene were examined, our results demonstrate that changes in methylation status as a result of storage conditions are possible, at least for individual genes. Our study thus points out that results of epigenetic studies could be distorted depending on the storage conditions and attention should be paid when comparing differentially stored samples. As it is conceivable that other genetic loci behave differently, future experiments need to be considered. According to the present state of knowledge, it appears that the best course to obtain reliable study results is to perform analyses directly after the collection of sample material. If this is not possible, early gDNA extraction, isolation of white blood cells or at least storage of EDTA blood with the addition of stabilizing agents at -80° C might be considered [11].

## Supporting information

S1 Supporting Information Primers, library preparation and detailed analyses of single CpG sites. (DOCX)

S1 Dataset DNA methylation results. (XLSX)
