# Supplementary material for: gDNA extraction yield and methylation status of blood samples are affected by long-term storage conditions
Source: PLoS One. 2018 Feb 7;13(2):e0192414. doi: 10.1371/journal.pone.0192414 (PMC5802893; doi:10.1371/journal.pone.0192414)
Supplement: S1 Supporting Information — (DOCX) [file pone.0192414.s001.docx]

**S1A Table. Primers.**

| **labeling** | **sequence (5' - 3')** | **bp** | **amplicon length^#^** | **T_M_ (°C)*** |
| --- | --- | --- | --- | --- |
| HIF3A.conv5.for1 | cgtatcgcctccctcgcgccatcagACGAGTGCGTGTTTTGGGTTTAATAAGGAATTTTATTT | 28 | 278 bp | 60.0 |
| HIF3A.conv5.for2 | cgtatcgcctccctcgcgccatcagACGCTCGACAGTTTTGGGTTTAATAAGGAATTTTATTT |  |  |  |
| HIF3A.conv5.for3 | cgtatcgcctccctcgcgccatcagAGACGCACTCGTTTTGGGTTTAATAAGGAATTTTATTT |  |  |  |
| HIF3A.conv5.for4 | cgtatcgcctccctcgcgccatcagAGCACTGTAGGTTTTGGGTTTAATAAGGAATTTTATTT |  |  |  |
| HIF3A.conv5.for5 | cgtatcgcctccctcgcgccatcagATCAGACACGGTTTTGGGTTTAATAAGGAATTTTATTT |  |  |  |
| HIF3A.conv5.for6 | cgtatcgcctccctcgcgccatcagATATCGCGAGGTTTTGGGTTTAATAAGGAATTTTATTT |  |  |  |
| HIF3A.conv5.for7 | cgtatcgcctccctcgcgccatcagCGTGTCTCTAGTTTTGGGTTTAATAAGGAATTTTATTT |  |  |  |
| HIF3A.conv5.for8 | cgtatcgcctccctcgcgccatcagCTCGCGTGTCGTTTTGGGTTTAATAAGGAATTTTATTT |  |  |  |
| HIF3A.conv5.for10 | cgtatcgcctccctcgcgccatcagTCTCTATGCGGTTTTGGGTTTAATAAGGAATTTTATTT |  |  |  |
| HIF3A.conv5.for11 | cgtatcgcctccctcgcgccatcagTGATACGTCTGTTTTGGGTTTAATAAGGAATTTTATTT |  |  |  |
| HIF3A.conv5.for13 | cgtatcgcctccctcgcgccatcagCATAGTAGTGGTTTTGGGTTTAATAAGGAATTTTATTT |  |  |  |
| HIF3A.conv5.for14 | cgtatcgcctccctcgcgccatcagCGAGAGATACGTTTTGGGTTTAATAAGGAATTTTATTT |  |  |  |
| HIF3A.conv5.rev1 | ctatgcgccttgccagcccgctcagACGAGTGCGTRATACAACCAAAACCCRAATAC | 22 |  | 60.0 |
| HIF3A.conv5.rev2 | ctatgcgccttgccagcccgctcagACGCTCGACARATACAACCAAAACCCRAATAC |  |  |  |
| HIF3A.conv5.rev3 | ctatgcgccttgccagcccgctcagAGACGCACTCRATACAACCAAAACCCRAATAC |  |  |  |
| HIF3A.conv5.rev4 | ctatgcgccttgccagcccgctcagAGCACTGTAGRATACAACCAAAACCCRAATAC |  |  |  |
| HIF3A.conv5.rev5 | ctatgcgccttgccagcccgctcagATCAGACACGRATACAACCAAAACCCRAATAC |  |  |  |
| HIF3A.conv5.rev6 | ctatgcgccttgccagcccgctcagATATCGCGAGRATACAACCAAAACCCRAATAC |  |  |  |
| HIF3A.conv5.rev7 | ctatgcgccttgccagcccgctcagCGTGTCTCTARATACAACCAAAACCCRAATAC |  |  |  |
| HIF3A.conv5.rev8 | ctatgcgccttgccagcccgctcagCTCGCGTGTCRATACAACCAAAACCCRAATAC |  |  |  |
| HIF3A.conv5.rev10 | ctatgcgccttgccagcccgctcagTCTCTATGCGRATACAACCAAAACCCRAATAC |  |  |  |
| HIF3A.conv5.rev11 | ctatgcgccttgccagcccgctcagTGATACGTCTRATACAACCAAAACCCRAATAC |  |  |  |
| HIF3A.conv5.rev13 | ctatgcgccttgccagcccgctcagCATAGTAGTGRATACAACCAAAACCCRAATAC |  |  |  |
| HIF3A.conv5.rev14 | ctatgcgccttgccagcccgctcagCGAGAGATACRATACAACCAAAACCCRAATAC |  |  |  |

*****melting temperatures [T_M_] were calculated by Primer3Plus Tool, T_M_ and base pairs according to the primers without MIDs and GS Junior-specific sequences; ^#^length of amplicon including MIDs and GS Junior-specific sequences; blue: GS Junior adaptor sequences, red: GS Junior recognition sequence, green: MIDs; R=A/G

**S1B Table. HIF3A library preparation ‑ polymerase chain reaction assay for thermocycler.**

| **PCR program** | | | |
| --- | --- | --- | --- |
| denaturing: 94 °C, 180 s  amplification: 30x [94 °C, 15 s; 56 °C, 45 s; 72 °C, 20 s]; 72 °C, 480 s; | | | |
| **Pipetting scheme** | | | |
| **reagents** | **volume (μl)** | **concentration** | **final concentration*** |
| H_2_O | 29 |  |  |
| Fast Start High Fidelity Reaction Buffer 2 (Roche) | 3.75 | 10x (within 18 nM MgCl_2_) | 1.06x |
| dNTP (Roche) | 0.75 | 10 mM | 212 μM |
| HIF3A.conv5.for | 0.75 | 25 μM | 530 nM |
| HIF3A.conv5.rev | 0.75 | 25 μM | 530 nM |
| FastStart HiFi Polymerase (Roche) | 0.375 | 5 U/μl | 1.875 U |
| total | 35.375 |  |  |
| DNA | 100 ng |  |  |


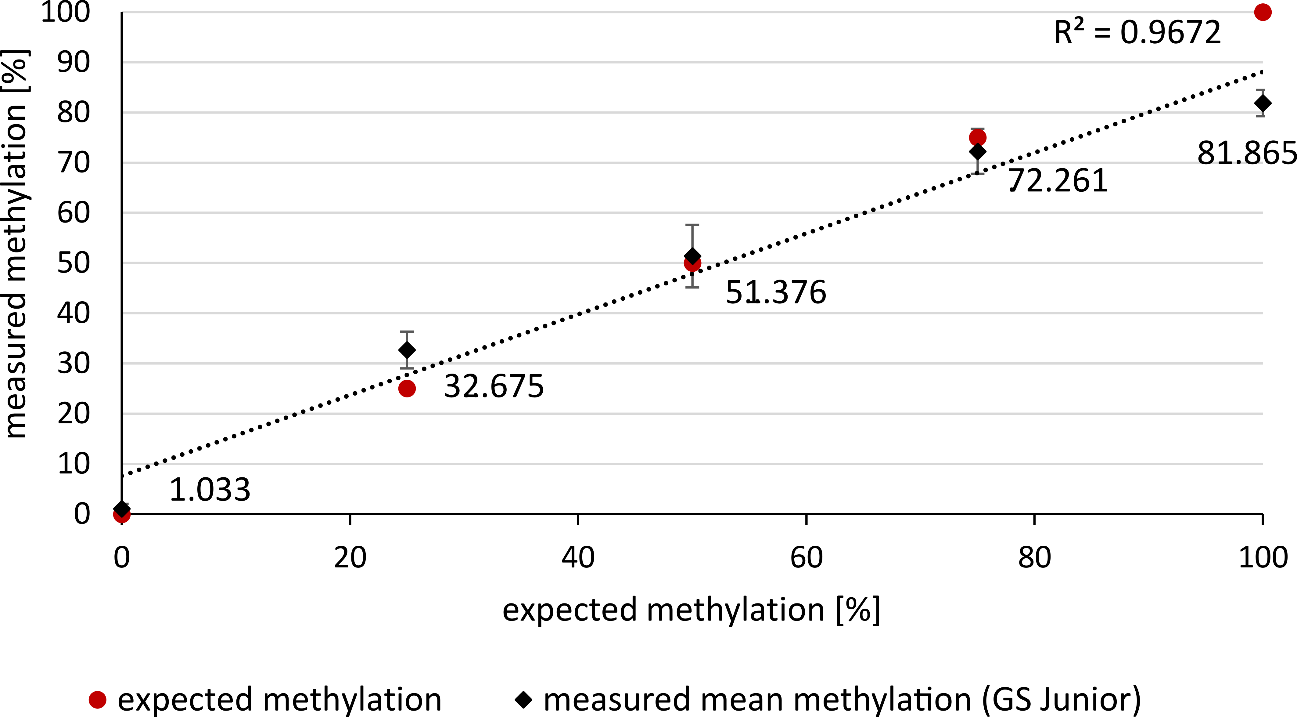


**S1 Fig. Standard measurements: Results of the measurements of DNA standards with known methylation values of 0, 25, 50, 75 and 100%.** Shown are the mean mesured methylation values and standard deviations for all CpG sites and line of regression (black) as well as the thoretical methylation values (red).

**S1C Table. Methylation levels of single CpG sites.** Overview of the mean values and mean absolute changes (dif abs) in methylation and standard deviations (SD) in relation to T_0_ for each measured CpG sites after storage for one, three and ten months as well as p-values.

| CpG site | storage time | T_0_ | 1 month | | | 3 months | | | 10 months | | |
| --- | --- | --- | --- | --- | --- | --- | --- | --- | --- | --- | --- |
|  | storage conditions | mean value ± SD | Mean value ± SD | dif abs ± SD | p | Mean value ± SD | dif abs ± SD | p | Mean value ± SD | dif abs ± SD | p |
| 1 | -70 °C | 14.61 ± 3.8 | 14.03 ± 4.1 | -0.57 ± 2.9 | 0.639 | 19.48 ± 3.6 | 4.86 ± 3.3 | 0.016 | 21.41 ± 3.6 | 6.80 ± 2.1 | 0.000 |
|  | -20 °C | 14.61 ± 3.8 | 15.56 ± 3.7 | 0.95 ± 2.2 | 0.205 | 15.91 ± 3.8 | 0.95 ± 2.2 | 0.682 | 20.33 ± 4.6 | 5.71 ± 2.4 | 0.000 |
|  | 2-8 °C | 14.61 ± 3.8 | 16.99 ±3.2 | 2.38 ± 2.6 | 0.019 | 18.17 ± 4.5 | 3.56 ± 2.5 | 0.002 | 15.90 ± 6.1 | 1.29 ± 4.6 | 0.397 |
|  | RT | 14.61 ± 3.8 | 15.06 ± 2.9 | 0.44 ± 3.4 | 0.690 | 16.90 ± 4.0 | 2.29 ± 3.4 | 0.063 | 20.59 ± 5.0 | 5.98 ± 4.8 | 0.003 |
|  | -70 °C/RT | 14.61 ± 3.8 | 15.76 ± 3.5 | 1.15 ± 3.2 | 0.290 | 15.86 ± 3.2 | 1.25 ± 2.1 | 0.091 | 18.61 ± 3.8 | 4.00 ± 3.5 | 0.005 |
|  |  |  |  |  |  |  |  |  |  |  |  |
| 2 | -70 °C | 28.32 ± 4.4 | 28.95 ± 4.7 | 0.63 ± 4.1 | 0.638 | 45.81 ± 4.8 | 17.5 ± 4.0 | 0.000 | 49.93 ± 4.2 | 21.6 ± 4.0 | 0.000 |
|  | -20 °C | 28.32 ± 4.4 | 31.27 ± 4.2 | 2.94 ± 3.8 | 0.035 | 40.91 ± 6.4 | 12.6 ± 4.5 | 0.000 | 47.88 ± 6.1 | 19.6 ± 5.5 | 0.000 |
|  | 2-8 °C | 28.32 ± 4.4 | 31.09 ± 5.1 | 2.76 ± 3.7 | 0.041 | 44.39 ± 6.5 | 16.1 ± 4.7 | 0.000 | 43.64 ± 8.5 | 15.3 ± 7.9 | 0.000 |
|  | RT | 28.32 ± 4.4 | 28.87 ± 5.3 | 0.55 ± 5.3 | 0.752 | 42.97 ± 6.8 | 14.6 ± 5.3 | 0.000 | 47.04 ± 6.0 | 18.7 ± 5.8 | 0.000 |
|  | -70 °C/RT | 28.32 ± 4.4 | 30.50 ± 4.4 | 2.17 ± 3.0 | 0.046 | 42.56 ± 6.4 | 14.2 ± 3.9 | 0.000 | 46.17 ± 6.5 | 17.8 ± 5.4 | 0.000 |
|  |  |  |  |  |  |  |  |  |  |  |  |
| 3 | -70 °C | 8.94 ± 2.7 | 8.50 ± 2.4 | -0.44 ± 2.5 | 0.589 | 9.40 ± 2.4 | 0.46 ± 2.5 | 0.570 | 13.86 ± 3.8 | 4.92 ± 2.9 | 0.000 |
|  | -20 °C | 8.94 ± 2.7 | 8.78 ± 2.3 | -0.16 ± 2.0 | 0.813 | 8.42 ± 2.8 | -0.51 ± 2.1 | 0.470 | 14.63 ± 4.8 | 5.69 ± 3.2 | 0.000 |
|  | 2-8 °C | 8.94 ± 2.7 | 8.99 ± 2.3 | 0.05 ± 1.6 | 0.924 | 9.86 ± 2.8 | 0.92 ± 2.3 | 0.233 | 13.28 ± 3.4 | 4.34 ± 2.5 | 0.000 |
|  | RT | 8.94 ± 2.7 | 9.17 ± 1.9 | 0.23 ± 2.9 | 0.808 | 8.97 ± 2.7 | 0.03 ± 2.5 | 0.970 | 14.19 ± 3.4 | 5.25 ± 3.1 | 0.000 |
|  | -70 °C/RT | 8.94 ± 2.7 | 9.66 ± 2.7 | 0.72 ± 2.8 | 0.430 | 8.46 ± 2.0 | -0.47 ± 1.7 | 0.409 | 14.32 ± 4.7 | 5.38 ± 3.0 | 0.000 |
|  |  |  |  |  |  |  |  |  |  |  |  |
| 4 | -70 °C | 3.90 ± 1.4 | 3.91 ± 0.8 | 0.005 ± 1.0 | 0.988 | 5.04 ± 1.1 | 1.1 ± 1.7 | 0.060 | 6.13 ± 1.0 | 2.2 ± 1.4 | 0.001 |
|  | -20 °C | 3.90 ± 1.4 | 4.40 ± 1.6 | 0.49 ± 1.1 | 0.201 | 3.63 ± 1.0 | -0.28 ± 1.0 | 0.395 | 5.02 ± 1.4 | 1.12 ± 1.6 | 0.056 |
|  | 2-8 °C | 3.90 ± 1.4 | 5.32 ± 1.6 | 1.41 ± 1.2 | 0.005 | 3.72 ± 0.88 | -0.18 ± 1.5 | 0.712 | 3.78 ± 1.6 | -0.12 ± 1.4 | 0.788 |
|  | RT | 3.90 ± 1.4 | 3.65 ± 1.2 | -026 ± 1.4 | 0.567 | 3.74 ± 0.86 | -0.16 ± 1.2 | 0.670 | 5.03 ± 1.9 | 1.13 ± 2.3 | 0.162 |
|  | -70 °C/RT | 3.90 ± 1.4 | 4.32 ± 1.8 | 0.42 ± 1.2 | 0.285 | 3.65 ± 0.81 | -0.25 ± 1.5 | 0.600 | 4.51 ± 1.3 | 0.61 ± 2.1 | 0.392 |
|  |  |  |  |  |  |  |  |  |  |  |  |
| 5 | -70 °C | 17.61 ± 5.8 | 16.52 ±5.3 | -1.09 ± 2.5 | 0.200 | 19.28 ± 4.9 | 1.67 ± 2.3 | 0.051 | 22.81 ± 6.2 | 5.20 ± 3.3 | 0.001 |
|  | -20 °C | 17.61 ± 5.8 | 18.68 ± 4.0 | 1.07 ± 2.3 | 0.180 | 17.23 ± 5.2 | -0.38 ± 2.1 | 0.579 | 22.46 ± 6.5 | 4.85 ± 2.4 | 0.000 |
|  | 2-8 °C | 17.61 ± 5.8 | 20.06 ± 5.4 | 2.45 ± 3.3 | 0.043 | 19.27 ± 5.6 | 1.66 ± 1.8 | 0.017 | 17.15 ± 6.4 | -0.46 ± 3.0 | 0.641 |
|  | RT | 17.61 ± 5.8 | 18.60 ± 4.7 | 0.99 ± 2.5 | 0.247 | 17.62 ± 5.3 | 0.01 ± 2.2 | 0.989 | 22.43 ± 6.3 | 4.82 ± 2.8 | 0.000 |
|  | -70 °C/RT | 17.61 ± 5.8 | 18.91 ± 6.0 | 1.30 ± 3.2 | 0.231 | 17.13 ± 4.5 | -0.48 ± 2.2 | 0.504 | 19.60 ± 5.9 | 1.99 ± 1.8 | 0.006 |
|  |  |  |  |  |  |  |  |  |  |  |  |
| 6 | -70 °C | 42.19 ± 5.6 | 41.12 ± 6.8 | -1.07 ± 3.9 | 0.402 | 42.77 ± 5.1 | 0.58 ± 3.8 | 0.638 | 55.24 ± 5.3 | 13.0 ± 2.0 | 0.000 |
|  | -20 °C | 42.19 ± 5.6 | 43.71 ± 5.4 | 1.52 ± 4.5 | 0.339 | 41.06 ± 6.4 | -1.13 ± 3.5 | 0.339 | 52.20 ± 5.3 | 10.0 ± 4.9 | 0.000 |
|  | 2-8 °C | 42.19 ± 5.6 | 48.89 ± 6.1 | 6.70 ± 4.3 | 0.001 | 42.36 ± 5.5 | 0.17 ± 2.5 | 0.838 | 46.07 ± 7.3 | 3.88 ± 5.0 | 0.037 |
|  | RT | 42.19 ± 5.6 | 44.26 ± 5.9 | 2.07 ± 3.4 | 0.083 | 39.03± 3.0 | -3.16 ± 2.2 | 0.001 | 52.89 ± 9.4 | 10.7 ± 4.6 | 0.000 |
|  | -70 °C/RT | 42.19 ± 5.6 | 43.10 ± 6.8 | 0.91 ± 4.2 | 0.511 | 40.64 ± 3.9 | -1.56 ± 3.8 | 0.228 | 52.20 ± 7.0 | 10.0 ± 3.0 | 0.000 |
|  |  |  |  |  |  |  |  |  |  |  |  |
| 7 | -70 °C | 17.82 ± 2.9 | 19.24 ± 4.3 | 1.42 ± 2.8 | 0.145 | 21.81 ± 4.5 | 3.99 ± 2.7 | 0.001 | 23.60 ± 5.3 | 5.78 ± 3.0 | 0.000 |
|  | -20 °C | 17.82 ± 2.9 | 21.07 ± 4.3 | 3.24 ± 3.1 | 0.009 | 18.84 ± 4.3 | 1.02 ± 1.7 | 0.093 | 23.60 ± 4.4 | 5.78 ± 2.5 | 0.000 |
|  | 2-8 °C | 17.82 ± 2.9 | 24.41 ± 6.1 | 6.59 ± 3.9 | 0.000 | 21.76 ± 4.2 | 3.93 ± 2.4 | 0.001 | 15.55 ± 3.5 | -2.28 ± 3.0 | 0.041 |
|  | RT | 17.82 ± 2.9 | 19.98 ± 5.5 | 2.16 ± 3.8 | 0.102 | 18.31 ± 4.8 | 0.49 ± 2.3 | 0.520 | 23.06 ± 4.1 | 5.24 ± 3.2 | 0.001 |
|  | -70 °C/RT | 17.82 ± 2.9 | 22.10 ± 4.0 | 4.28 ± 2.6 | 0.001 | 19.30 ± 3.4 | 1.48 ± 2.2 | 0.052 | 21.15 ± 4.5 | 3.32 ± 2.6 | 0.003 |
|  |  |  |  |  |  |  |  |  |  |  |  |
| 8 | -70 °C | 71.59 ± 4.7 | 71.66 ± 6.3 | 0.08 ± 2.7 | 0.931 | 74.8 ± 6.3 | 3.17 ± 3.1 | 0.010 | 76.58 ± 5.4 | 5.00 ± 3.0 | 0.001 |
|  | -20 °C | 71.59 ± 4.7 | 73.58 ± 5.1 | 2.00 ± 3.6 | 0.111 | 72.73 ± 6.7 | 1.14 ± 3.1 | 0.270 | 75.74 ± 5.0 | 4.15 ± 3.0 | 0.002 |
|  | 2-8 °C | 71.59 ± 4.7 | 77.24 ± 6.2 | 5.66 ± 3.6 | 0.001 | 74.01 ± 5.5 | 2.42 ± 3.0 | 0.031 | 70.92 ± 6.7 | -0.66 ± 3.9 | 0.602 |
|  | RT | 71.59 ± 4.7 | 72.54 ± 7.1 | 0.96 ± 3.2 | 0.374 | 71.86 ± 6.5 | 0.28 ± 3.0 | 0.776 | 76.38 ± 6.2 | 4.79 ± 4.9 | 0.013 |
|  | -70 °C/RT | 71.59 ± 4.7 | 72.89 ± 6.2 | 1.31 ± 3.6 | 0.285 | 71.74 ± 4.7 | 0.15 ± 2.5 | 0.853 | 75.91 ± 5.8 | 4.32 ± 2.9 | 0.001 |
|  |  |  |  |  |  |  |  |  |  |  |  |
| 9 | -70 °C | 46.86 ± 5.7 | 46.54 ± 5.9 | -0.32 ± 1.9 | 0.607 | 52.15 ± 6.2 | 5.29 ± 2.1 | 0.000 | 59.06 ± 4.7 | 12.2 ± 2.8 | 0.000 |
|  | -20 °C | 46.86 ± 5.7 | 49.28 ± 5.8 | 2.41 ± 4.3 | 0.110 | 48.11 ± 6.7 | 1.25 ±2.1 | 0.096 | 57.42 ± 6.2 | 10.6 ± 2.8 | 0.000 |
|  | 2-8 °C | 46.86 ± 5.7 | 54.63 ± 6.1 | 7.78 ± 3.5 | 0.000 | 50.14 ± 5.9 | 3.28 ± 2.8 | 0.005 | 47.71 ± 6.9 | 0.85 ± 5.0 | 0.603 |
|  | RT | 46.86 ± 5.7 | 48.32 ± 6.7 | 1.46 ± 4.2 | 0.304 | 46.90 ± 7.9 | 0.04 ± 3.8 | 0.973 | 56.92 ± 7.1 | 10.1 ± 5.9 | 0.000 |
|  | -70 °C/RT | 46.86 ± 5.7 | 48.51 ± 5.1 | 1.65 ± 4.0 | 0.224 | 48.53 ± 4.3 | 1.67 ± 2.3 | 0.050 | 56.48 ± 7.5 | 9.63 ± 2.7 | 0.000 |
|  |  |  |  |  |  |  |  |  |  |  |  |
| 10 | -70 °C | 42.92 ± 7.2 | 42.50 ± 8.2 | -0.42 ± 2.8 | 0.651 | 46.31 ± 7.0 | 3.39 ± 2.8 | 0.004 | 51.36 ± 6.7 | 8.44 ± 3.6 | 0.000 |
|  | -20 °C | 42.92 ± 7.2 | 44.53 ± 6.8 | 1.61 ± 3.7 | 0.205 | 44.06 ± 8.3 | 1.14 ± 3.3 | 0.300 | 49.44 ± 6.3 | 6.52 ± 3.1 | 0.000 |
|  | 2-8 °C | 42.92 ± 7.2 | 47.43 ± 7.1 | 4.51 ± 3.3 | 0.002 | 44.91 ± 7.3 | 4.99 ± 3.7 | 0.119 | 44.05 ± 10.2 | 1.13 ± 7.2 | 0.632 |
|  | RT | 42.92 ± 7.2 | 44.39 ± 6.5 | 1.47 ± 3.1 | 0.170 | 41.77 ± 8.2 | -0.15 ± 3.1 | 0.884 | 49.59 ± 6.3 | 6.66 ± 4.7 | 0.002 |
|  | -70 °C/RT | 42.92 ± 7.2 | 4.26 ± 7.3 | -0.67 ± 2.8 | 0.478 | 42.76 ± 6.5 | -0.16 ± 2.5 | 0.845 | 48.02 ± 9.1 | 5.10 ± 3.9 | 0.003 |
|  |  |  |  |  |  |  |  |  |  |  |  |

**S1D Table. Comparison of percentage increase in methylation of individual CpG sites.** Mean absolute methylation levels at starting point (T_0_) and after 10 month of storage at ‑70 °C (T_10_) as well as mean percentage increase in methylation for individual CpG sites. Other storage conditions show similar outcomes. (Data not shown.)

| CpG site | methylation level T_0_ ± SD | methylation level T_10_ ± SD | percentage increase in methylation [%] ± SD |
| --- | --- | --- | --- |
| 4 | 3.90 ± 1.4 | 6.13 ± 1.0 | 75.8 ± 63.7 |
| 3 | 8.94 ± 2.7 | 13.86 ± 3.8 | 61.7 ± 43.7 |
| 1 | 14.6 ± 3.8 | 21.41 ± 3.6 | 51.0 ± 25.1 |
| 5 | 17.6 ± 5.8 | 22.81 ± 6.2 | 34.6 ± 28.9 |
| 7 | 17.8 ± 2.9 | 23.60 ± 5.3 | 31.6 ± 14.3 |
| 2 | 28.3 ± 4.4 | 49.93 ± 4.2 | 78.8 ± 22.4 |
| 6 | 42.2 ± 5.6 | 55.24 ± 5.3 | 31.6 ± 7.6 |
| 10 | 42.9 ± 7.2 | 51.36 ± 6.7 | 20.7 ± 11.2 |
| 9 | 46.9 ± 5.7 | 59.06 ± 4.7 | 26.8 ± 8.6 |
| 8 | 71.6 ± 4.7 | 76.58 ± 5.4 | 7.03 ± 4.6 |
